# The Potential Vector Competence and Overwintering of West Nile Virus in Vector *Aedes Albopictus* in China

**DOI:** 10.3389/fmicb.2022.888751

**Published:** 2022-06-02

**Authors:** Ying-mei Zhang, Xiao-xia Guo, Shu-fang Jiang, Chun-xiao Li, Dan Xing, Heng-duan Zhang, Yan-de Dong, Tong-yan Zhao

**Affiliations:** ^1^State Key Laboratory of Pathogen and Biosecurity, Beijing Institute of Microbiology and Epidemiology, Beijing, China; ^2^First Medical Center, Chinese People’s Liberations Army (PLA) General Hospital, Beijing, China

**Keywords:** West Nile virus, *Aedes Albopictus*, infection, transmission, overwinter, diapausing

## Abstract

West Nile virus (WNV) is an arbovirus, which causes widespread zoonotic disease globally. In China, it was first isolated in Jiashi County, Kashgar Region, Xinjiang in 2011. Determining the vector competence of WNV infection has important implications for the control of disease outbreaks. Four geographical strains of *Aedes Albopictus* (*Ae. Albopictus*) in China were allowed to feed on artificial infectious blood meal with WNV to determine the infection and transmission rate. The results indicated that four strains of *Ae. Albopictus* mosquitoes could infect and transmit WNV to 1- to 3-day-old Leghorn chickens. The infection rates of different strains were ranged from 16.7 to 60.0% and were statistically different (χ^2^ = 12.81, *p* < 0.05). The highest infection rate was obtained from the Shanghai strain (60.0%). The transmission rates of *Ae. Albopictus* Shanghai, Guangzhou, Beijing, and Chengdu strains were 28.6, 15.2, 13.3, and 6.7%, respectively. Furtherly, the results reveal that *Ae. Albopictus* Beijing strain infected orally can transmit WNV transovarially even the eggs are induced diapausing. The study confirmed that WNV could survive in the diapause eggs of *Ae. Albopictus* and could be transmitted to progeny after diapause termination. This is of great significance for clarifying that the WNV maintains its natural circulation in harsh environments through inter-epidemic seasons.

## Introduction

West Nile virus (WNV) is an important zoonotic arbovirus of the family *Flaviviridae* (Heinz et al., 2000). It was first isolated in Africa in 1937 and circulated in a transmission cycle involving mosquitoes and birds (Gray and Webb, 2014; Chancey et al., 2015). West Nile fever is a global emerging disease. There are a large variety of habitats for migrating birds and resident birds in mainland China. Infectious migrating birds likely were important in WNV amplification (Julian et al., 2002). Therefore, there would be an endemic risk of WNV in China. Serological results for WNV infection in birds and humans suggested that enzootic transmission existed in China (Li et al., 2013). The human infections of WNV were reported in 2013 in Xinjiang (Lu et al., 2014; Cao et al., 2017, 2019). WNV was isolated from mosquitoes firstly in Xinjiang in western China (Lu et al., 2014; Zhang et al., 2021). Therefore, increasing geographic distribution and circulation of WNV infection in mainland China should be noted.

West Nile virus has been isolated in 43 different ornithophilic *Culex* mosquito species that include *Aedes* and *Ochlerotatus* (Centers for Disease Control and Prevention [CDC], 2003). In China, four *Culex* mosquitoes were confirmed to be capable of WNV infection and transmission (Jiang et al., 2010). *Aedes Albopictus* (*Ae. Albopictus*) Skuse is widely distributed in mainland China with a range extending from south to Hainan Island, north to Shenyang in Liaoning Province, west to Tianshui and Longnan in Gansu Province, and southwest to Motuo in Tibet in China (Lu, 1990). Viral isolation and vector competence studies have established the efficiency of *Ae. Albopictus* in the transmission of more than 20 arboviruses (Paupy et al., 2009). *Ae. Albopictus* is considered a classic bridge vector between zoonotic arboviruses and humans due to its opportunistic feeding behavior (Paupy et al., 2009; Fortuna et al., 2015).

The study on the susceptibility of three North American *Ae. Albopictus* strains (Frederick County, Maryland, FRED strain; Cheverly, MD, CHEV strain; Chambers and Liberty counties, Texas, TAMU strain) to WNV revealed that the susceptibility of the strain from a Hawaiian source (OAHU strain) and TAMU was similar, but different from the FRED and CHEV strains (Sardelis et al., 2002). Currently, little is known about whether *Ae. Albopictus* plays a role in WNV transmission in China. Due to the widespread distribution in China, along with its opportunistic feeding behavior, ecological adaptability, and propensity, the aim of the present study was to evaluate the vector competence of *Ae. Albopictus*. The study presents the oral susceptibility and vector competence of four geographical strains of *Ae. Albopictus* to WNV. Four *Ae. Albopictus* strains are crossing subtropical and temperate regions of China. The results would deepen to the understanding of WNV infection and transmission in different mosquito strains. It would enhance the system of surveillance and control of vectors to prevent the outbreak of WNV in China.

*Aedes Albopictus* egg undergoes facultative diapause as the mechanism for surviving unfavorable environments, such as winter or arid and frigid environments (Hong et al., 1971). Therefore, the egg diapause mechanism may contribute to arbovirus preservation. A previous study has confirmed that the Japanese encephalitis virus can survive in dried infected *Ae. Albopictus* eggs for 2 months (Rosen et al., 1978). Additionally, dengue virus 2 was detected in diapause eggs of *Ae. Albopictus* (Guo et al., 2007) and transmitted to progeny after diapause termination (Guo et al., 2004). Diapausing behavior may provide an important mechanism for the maintenance of arbovirus under adverse climatic conditions. This work is aimed to confirm the possibility of WNV surviving in the cold season and playing a possible role in an endemic cycle in the next year.

## Materials and Methods

### Ethics Statement

The study was conducted in the Animal Biosafety Level 3 (ABSL-3) facility. All of the experimental protocols involving animals were approved by the Laboratory Animal Center of the State Key Laboratory of Pathogen and Biosecurity, Beijing Institute of Microbiology, and Epidemiology Institutional Animal Care and Use Committee (IACUC, the permit number is BIME 2011-2009). The animals were performed in strict accordance with the recommendations of the Guide for the Care and Use of Laboratory Animals of the National Institutes of Health.

### Mosquitoes

Four geographical strains of *Ae. Albopictus* were originally collected in Guangzhou city (GPS location: 23°08′N and 113°14′W) in 1996, Shanghai city (GPS location: 31°15′N and 121°30′W) in 1995, Chengdu city (GPS location: 30°05′N and 102°54′W) in 1996, and Beijing city (GPS location: 39°56′N and 116°20′W) in 1997, respectively. Mosquitoes were maintained at 25 ± 1°C and 75 ± 5% relative humidity (RH) under a 14-h light/10-h dark (LD) photoperiod. F30 generation of *Ae. Albopictus* mosquitoes was used in this study. In addition, the Guangzhou strain and Beijing strain of *Ae. Albopictus* were collected in the wild in 2004 and 2005, separately. Mosquitoes were domesticated to F3 generation in the laboratory for transmission efficiency evaluation.

### Virus Strain and Cell Line

The WNV strain (GenBank AY490240) was provided by the Microbial Culture Collection Center, Beijing Institute of Microbiology and Epidemiology. It had been passaged six times in Vero cells. Viral titer was 10^7^.^0^–10^7^.^5^ PFU/ml. Vero cells were cultivated in Dulbecco’s modified Eagle’s medium (DMEM) (GIBCO™, Invitrogen, Beijing, China) containing 10% fetal bovine serum (FBS) and placed in an incubator of 37°C and 5% CO_2_.

### Mosquito Infection

Three- to five-day-old female mosquitoes were deprived of glucose solution and water for 24 h prior to infection. The infectious blood meal was composed of 1:1:1 mouse blood and virus and an 8% sugar solution. The viral titer of infectious blood meals was 10^6^.^7^–10^6^.^9^ PFU/ml. Mosquitoes were offered a WNV infectious blood meal for 1 h using a Hemotek membrane feeding system housed in a feeding chamber that was constantly warmed to 37°C. One hundred and fifty engorged female mosquitoes were selected under CO_2_ sedation for each geographical strain and transferred to three small cages (50 mosquitoes/cage), respectively. Engorged mosquitoes were reared under CO_2_ sedation and incubated under 29 ± 1°C and 80 ± 5% RH with a 14:10 (light:dark) photocycle.

### Virus Assay and Reverse Transcription-Polymerase Chain Reaction

West Nile virus-exposed samples were detected by reverse transcription-polymerase chain reaction (RT-PCR). The PCR primers included a pair of universal primers, Primer 1 (P1): P1:5′-TTG TGT TGG CTC TCT TGG CGT TCTT and Primer 2 (P2): 5′-CAG CCG ACA GCA CTG GAC ATT CATA. The RNA extraction was performed using TRIzol reagent (Invitrogen) according to the recommendations of manufacturers. Viral RNA extract was transcribed into cDNA using RNA PCR Kit (AMV) version 2.1 [Takara Biotechnology (Dalian) Co., Ltd., Shiga, Japan]. The resulting cDNA was used as a template in the subsequent PCR reaction. The PCR amplifications were carried out through 45 cycles at 94°C for 30 s, 58°C for 30 s, 72°C for 1 min, and final extension at 72°C for 7 min. Furtherly, some WNV-exposed samples were selected randomly to identify by C6/36 culture isolation. Virus isolation was processed as described in Jiang et al.’s (2006) study. That is, the supernatants of infected mosquitoes or Leghorn chicken blood were passed through a filter with pores of 0.22 μm in diameter and inoculated into duplicate wells of C6/36 cell microplate cultures. After 1.5 h incubation at 37°C, cells were maintained at 37°C in DMEM supplemented with penicillin (100 U/ml), streptomycin (100 μg/ml), and 5% FBS for 7 days.

### Transmission Experiment of Four Geographic Strains of *Aedes Albopictus* to West Nile Virus

Mosquitoes were deprived of glucose solution and water for 24 h after a 14-day incubation period. One mosquito was allowed to feed on one 1- to 3-day-old Leghorn chicken individually for 30 min. After sucking blood, mosquitoes were killed by freezing at 20°C and were ground individually in 1.0 ml of mosquito diluents to test for the presence of the virus by RT-PCR. Then RNA positive samples were inoculated into C6/36 cells to isolate WNV. On day 2 post-sucking, the chicken blood was collected and serum was separated. The serum was then used to detect viral RNA by RT-PCR and to isolate WNV by inoculation into C6/36 cells.

The infection rate describes the number of mosquitoes infected with WNV in their body in relation to the total number of mosquitoes examined. The transmission rate was calculated as the ratio of the number of mosquitoes that can transmit the virus by biting to the total number of infected mosquitoes (Goddard et al., 2002; Tiawsirisup et al., 2005). The transmission efficiency is the percentage of WNV-positive mosquitoes that can transmit the virus by biting in relation to the total number of engorged mosquitoes.

### Mosquito Egg Collection

**FIGURE 1:**
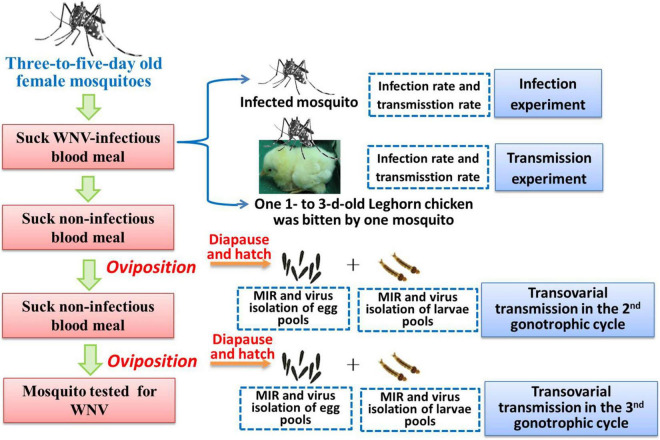
A schematic representation of the experimental design.

Eggs from the second and third gonotrophic cycles of *Ae. Albopictus* Beijing strain were used in this study. Filter paper used as an oviposition substrate was placed in a small container of water after blood meal. After 3 days, filter papers were removed and kept in humid environment for 48 h. Then, eggs were held in desiccators at 25 ± 1°C, 90% RH with a daily photoperiod of 14-h light/10-h dark (LD) for 6 days to permit embryonation (Hanson and Craig, 1994). A schematic representation of the experimental design and infection, transmission, and diapause induction is shown in Figure 1.

### Diapause Induction and West Nile Virus Detection

Following embryonation, eggs were placed into a light cultivation chamber (HPG-280H) (Guangdong Medical Instrument Factory Co., Ltd.) and subjected to a 10-h light/14-h dark (LD) photoperiod and 75% RH was controlled constantly with a supersaturated salt solution (Winston and Bates, 1960). The temperature was decreased 5°C every 2 days from 25 to 10°C. At last, eggs were held at a daily photoperiod of 8-h light/16-h dark (LD) and thermoperiod of 10°C:4°C (LD) and 50% RH for 6 weeks. The diapause induction procedure for uninfected eggs was the same. The control group not in diapause (non-diapause) was maintained at 25 ± 1°C and 14-h light/10-h dark (LD) photoperiod and 75% RH.

Reverse transcription-PCR and virus isolation in C6/36 cells were used to detect WNV in diapausing eggs from the second and third gonotrophic cycles of infected *Ae. Albopictus*. Pools of diapausing eggs (200 eggs per pool) were detected. Minimum infection rates (MIRs) were calculated using Centers for Disease Control and Prevention (CDC) protocols.^1^

### Diapausing Egg Hatch

Diapausing eggs of *Ae. Albopictus* Beijing strain were submerged in deoxygenated solution (500 mg larval food was added into 500 ml water for 24 h, the dissolved oxygen concentration was about 0.3–0.5 ppm) to terminate diapause. After 24 h, the number of larvae was counted. All samples then were transferred to non-diapausing conditions [25 ± 1°C and 14-h light/10-h dark (LD)] and hatch was attempted weekly. Unhatched eggs were bleached to clear the chorion to determine embryonation. The bleach solution consisted of 40 g sodium hypochlorite, 10 ml acetic acid, and 1 L of water (Hanson and Craig, 1994). Pools of F1 generation larvae from diapausing and non-diapausing eggs were detected by RT-PCR and virus isolation with C6/36. MIRs were calculated.

### Statistical Analysis

The significance of any differences in infection and transmission rates between four geographical strains of *Ae. Albopictus* were tested with Chi-square and Fisher’s exact tests implemented in the SPSS (GraphPad Software, San Diego, CA, United States) Version 10.0. Values of *p* < 0.05 were considered significant.

## Results

### Oral Susceptibility of Four Geographical Strains of *Aedes Albopictus*

Four geographical strains of *Ae. Albopictus* were susceptible to infection with WNV after imbibing the virus dose of 10^6^.^7^–10^6^.^9^ PFU/ml. The infection rates of four different geographical strains of *Ae. Albopictus* are shown in Table 1. The infection rates of different strains tested in the study were ranged from 16.7 to 60.0%. There were significant differences in infection rates between the four strains (χ^2^ = 12.81, *p* < 0.05). The highest infection rate was found in the Shanghai strain, which was 60.0%. There was no significant difference between the *Ae. Albopictus* 30 generations and 3 generations mosquito (χ^2^ = 2.73, *p* > 0.05) of Guangzhou and Beijing strains. Four RNA-positive samples were inoculated into C6/36 cells to isolate WNV. Cytopathic effect (CPE) was observed on the fifth day after inoculation.

**TABLE 1:** Infection rate and transmission rate and transmission efficiency of four geographical *Aedes Albopictus* strains after oral exposure to West Nile virus (WNV).

Strains	Generations	No. tested mosquito	No. infected mosquito	Infection rate (%)	No. infected Leghorn chicken	Transmission rate (%)	Transmission efficiency (%)
Shanghai	30	35	21	60.0 (21/35)	10	47.6 (10/21)	28.6 (10/35)
Chengdu	30	30	5	17.9 (5/28)	2	40 (2/5)	7.1 (2/28)
Guangzhou	30	33	11	36.7 (11/30)	5	45.5 (5/11)	16.7 (5/30)
	3	30	7	23.3 (7/30)	3	42.9 (3/7)	10 (3/30)
Beijing	30	30	9	30.0 (9/30)	4	44.4 (4/9)	13.3 (4/30)
	3	30	5	16.7 (5/30)	2	40 (2/5)	6.7 (2/30)

### Transmission Experiment of Four Geographical Strains of *Aedes Albopictus* to West Nile Virus

Four geographical strains of *Ae. Albopictus* were able to transmit WNV to 1- to 3-day-old Leghorn chicken by biting. Transmission rates varied from 40.0 to 47.6% (Table 1). There were no significant differences in transmission rates between the different strains (χ^2^ = 4.49, *p* > 0.05). However, there were significant differences in transmission efficiency between the four geographical strains of *Ae. Albopictus* (χ^2^ = 103.2, *p* < 0.05). The transmission efficiency of the Shanghai and Chengdu strains was significantly different (χ^2^ = 5.149, *p* < 0.05). The Leghorn chickens were bled on the second day for virus assay by RT-PCR. Leghorn chickens had viral RNA in their blood. The serum of three Leghorn chickens was inoculated into C6/36 cells. CPE was observed on the seventh day after inoculation.

### West Nile Virus Detection in Diapausing Eggs

Pools of diapausing and non-diapausing eggs (200 eggs per pool) from the second and third gonotrophic cycles were tested by RT-PCR. An amplification product of the predicted size of 408 bp was evident in diapausing eggs. All RNA-positive homogenates of diapausing and non-diapausing eggs were inoculated in C6/36 cells for virus isolation. CPE was observed 4-day post-inoculation in diapausing and non-diapausing eggs. Both the PCR amplification results and virus isolation in C6/36 cells confirmed that WNV infected diapausing eggs (Table 2).

**TABLE 2:** West Nile virus (WNV) detection in diapausing eggs of *Ae. Albopictus* Beijing strain.

Eggs	Gonotrophic cycle	No. pools (200 eggs/pool)	WNV-positive pools	MIR^a^
Diapause	Second	6	1	1:1200
	Third	8	2	1:800
Non-diapause	Second	5	1	1:1000
	Third	6	2	1:600

*^a^Minimum infection rate (MIR) is the ratio of the number of positive pools to the total number of individuals (eggs) tested.*

### West Nile Virus Detection in F1 Generation Larvae

West Nile virus was detected in F1 generation larvae from diapausing eggs. An amplification product of the predicted size of 408 bp was evident in F1 generation larvae. RNA-positive samples were inoculated into C6/36 cells to isolate the virus. CPE was observed 6-day post-inoculation in F1 generation larvae. The results showed that WNV can be detected in the larvae hatched from diapausing eggs. The MIR of F1 generation larvae from diapausing eggs was 1:1180 in the second gonotrophic cycle and 1:1120 in the third gonotrophic cycle (Table 3). There were no significant differences in the MIR of F1 generation larvae between diapausing and non-diapausing eggs (1:960 and 1:1120, respectively; *p* > 0.05). There was no significant difference in the MIR in larvae between the second and third gonotrophic cycles (*p* > 0.05).

**TABLE 3:** West Nile virus (WNV) detection in F1 generation larvae hatched from diapausing eggs of *Aedes Albopictus* (*Ae. Albopictus*) Beijing strain.

Mosquito stage	Gonotrophic cycle	No. pools (total number)	WNV-positive pools	MIR^a^
Larvae from diapausing eggs	Second	13 (1180)	1	1:1180
	Third	12 (1120)	2	1:1120
Larvae from non-diapausing eggs	Second	10 (960)	1	1:960
	Third	13 (1300)	2	1:650

*^a^Minimum infection rate (MIR) is the ratio of the number of positive pools to the total number of individuals (larvae) tested.*

## Discussion

West Nile virus is a re-emerging infectious disease and expands its geographic range in Europe and in other parts of the world (Weaver and Reisen, 2010; Koray et al., 2014). In mainland China, the first WNV isolation from mosquitos in Xinjiang and the prevalence of fever of viral encephalitis caused by WNV infection has been recorded (Li et al., 2013; Cao et al., 2017, 2019). Therefore, monitoring WNV infection is important for public health in China.

Vector competence refers to the intrinsic permissiveness of an arthropod vector to infection, replication, and transmission of the virus. In the present study, the infection rate, transmission rate, and transmission efficiency of WNV in four geographical strains of *Ae. Albopictus* from China were evaluated. Our results showed that four geographical strains of *Ae. Albopictus* were susceptible to infect with WNV after imbibing the virus dose of 10^6^.^7^–10^6^.^9^ PFU/ml. In nature, wild birds (e.g., crows) can produce viral titers of 10^8^ PFU/ml (Cao et al., 2019), and the viral titers 10^6^.^7^–10^6^.^9^ PFU/ml are used in this study should be representative of the dose exposed to mosquitoes in nature. Therefore, *Ae. Albopictus* is able to infect WNV by bloodsucking. The Leghorn chicken bitten by infectious mosquitoes can be infected with WNV, which is the direct evidence for *Ae. Albopictus* as a potential vector. Current studies mostly agree that the main vector of WNV is *Culex*, most *Culex* species are bloodthirsty for bird and play an important role in the WNV maintenance and spread in nature. However, the habit of bloodthirsty birds may reduce the danger of transmitting the virus to mammals, such as humans and horses. The opportunistic feeding behavior of *Ae. Albopictus* could transmit WNV from the amplifying bird hosts to mammalians and act as “bridge vectors.” Currently, both virus isolation in the field and transmission experiments in the laboratory indicate that *Ae. Albopictus* is able to transmit WNV and is a possible bridge vector for WNV (Turell et al., 2001; Sardelis et al., 2002). Once the number of virus-infected birds and the population of *Aedes* mosquitoes increased, the danger of WNV transmission from the natural cycle of mosquito to bird to susceptible animals, such as humans and horses, is heightened.

The latitude of *Ae. Albopictus* distribution is also extremely wide, being present in regions south of 41°N and with the highest density of south 32°N (Lu, 1990). Different geographic strains of *Ae. Albopictus* have been shown to vary in their susceptibility to the dengue virus (Woodring et al., 1996). The present study found that four geographic *Ae. Albopictus* strains from both south and north of China were able to infect and spread WNV, with significantly higher transmission rates for the Shanghai strain, and a lower transmission rate for the Beijing, Chengdu, Guangzhou strains. There were significant differences in transmission efficiency between the four geographical strains of *Ae. Albopictus* (*p* < 0.05). *Ae. Albopictus* should therefore be considered a WNV vector in regions of China. The results indicated that *Ae. Albopictus* exhibits geographic variation in vector competence to WNV. A further study on the genetic basis of vector infection and the competence of different geographic strains to transmit WNV should be conducted. In addition, statistical analysis indicated that there were no significant differences between a laboratory strain and field strain on WNV susceptibility (*p* > 0.05). The results showed that there was no significant effect of generation on susceptibility to WNV.

Egg diapause is an essential condition for *Ae. Albopictus* to overwinter and/or survive in harsh environments through inter-epidemic seasons. Eggs from two gonotrophic cycles of WNV-infected mosquitoes were collected and inducted diapause. The present study confirmed that WNV can live in the overwintering diapause eggs of *Ae. Albopictus* and transmit to the offspring, which plays a positive role in maintaining the survival and long-distance transmission of the virus under adverse conditions. At the same time, *Ae. Albopictus* is widely distributed in China, and China’s climate conditions are complex. Most areas in the north are cold and dry in winter, and the RH is basically maintained at about 50%, while most areas in the south are cold and humid in winter, and the RH is maintained at about 75%. The results of this study show that WNV can survive in the diapause eggs of *Ae. Albopictus* at 50% RH and transmit to the offspring, which suggests that WNV may maintain its transmission cycle in nature through the diapause eggs of *Ae. Albopictus* in both cold and dry areas. This may be of great significance in the natural epidemic of diseases. Accordingly, more attention should be paid to *Ae. Albopictus* eggs in vector control for WNV outbreak management at temperate latitudes.

## Data Availability Statement

The original contributions presented in the study are included in the article/supplementary material, further inquiries can be directed to the corresponding author.

## Ethics Statement

The animal study was reviewed and approved by the Laboratory Animal Center of the State Key Laboratory of Pathogen and Biosecurity, Beijing Institute of Microbiology and Epidemiology Institutional Animal Care and Use Committee (IACUC, the permit number is BIME 2011-2009).

## Author Contributions

T-YZ designed the study. Y-MZ, S-FJ, DX, and H-DZ carried out the data acquisition and analysis. X-XG wrote the manuscript. C-XL and Y-DD supervised the study. All authors reviewed the manuscript.

## Conflict of Interest

The authors declare that the research was conducted in the absence of any commercial or financial relationships that could be construed as a potential conflict of interest.

## Publisher’s Note

All claims expressed in this article are solely those of the authors and do not necessarily represent those of their affiliated organizations, or those of the publisher, the editors and the reviewers. Any product that may be evaluated in this article, or claim that may be made by its manufacturer, is not guaranteed or endorsed by the publisher.

## Footnotes

1
https://www.cdc.gov/westnile/resourcepages/mosqsurvsoft.html
